# Crystal structure of ochraceolide A isolated from *Elaeodendron trichotomum* (Turcz.) Lundell

**DOI:** 10.1107/S2056989017012816

**Published:** 2017-09-15

**Authors:** Angel D. Herrera-España, Gonzalo J. Mena-Rejón, Simón Hernández-Ortega, Leovigildo Quijano, Gumersindo Mirón-López

**Affiliations:** a Facultad de Química, Universidad Autónoma de Yucatán, Calle 43 No. 613, Col. Inalámbrica, 97069, Mérida, Yucatán, Mexico; b Instituto de Química, Universidad Nacional Autónoma de México, Circuito, Exterior, Ciudad Universitaria, 04510, Mexico City, Mexico

**Keywords:** crystal structure, ochraceolide A, triterpene lactone, *Elaeodendron trichotomum*

## Abstract

The title compound, C_30_H_44_O_3_ [systematic name: 6a*R*,6 b*R*,8a*S*,9a*R*,12a*R*,14b*R*)-4,4,6a,6;b,8a,14b-hexa­methyl-12-methyl­eneicosa­hydro-3*H*-phenanthro[1′,2′:6,7]indeno­[2,1-*b*]furan-3,11(2*H*)-dione], is a triterpene lactone, which was isolated from di­chloro­methane extract of *Elaeodendron trichotomum* (Turcz.) Lundell (celastraceae) stem bark. The compound has a lupane skeleton and consists of four fused six-membered rings and two five-membered rings. In the crystal, mol­ecules are linked by weak C—H⋯O hydrogen bonds into a three-dimensional network. The configuration of ochraceolide A was proposed based on analogue compounds which belong to the lupane type.

## Abstract 2

The crystal structure of the triterpene lactone ochraceolide A (3-oxolup-20 (29)-en-30,21α-olide) isolated from *Elaeodendron trichotomum* (Turcz.) Lundell is reported.

## Chemical context   

Ochraceolides A–E are a group of cytotoxic lupane γ-lactones isolated from the Celastraceae family. Ochraceolide A was firstly isolated from *Kokoona ochracea* (Elm.) Merril stem bark (Ngassapa *et al.*, 1991 ▸) and afterwards from *Lophopetalum wallichii* (Sturm *et al.*, 1996 ▸) and *Cassine xylocarpa* (Callies *et al.*, 2015 ▸). The title compound has shown significant cytotoxic activity against murine lymphocytic leukemia cells (P-388) with an ED_50_ of 0.6 µ*M*; human oral epidermoid carcinoma (KB-3) with an ED_50_ of 6.0 µ*M*; and hormone-dependent breast cancer with an ED_50_ of 9.9 µ*M* (Ngassapa *et al.*, 1991 ▸; Sturm *et al.*, 1996 ▸). In the same way, this compound has exhibited significant inhibitory activity in the FPTase assay with an IC_50_ of 2.2 µ*M* (Sturm *et al.*, 1996 ▸) and inhibitory effects of human immunodeficiency virus type 1 replication with an IC_50_ of 39.0 µ*M* (Callies *et al.*, 2015 ▸). Ochraceolide A is part of the structure of the Diels–Alder adduct (*i.e.* celastroidine A or volubilide) isolated from *Hippocratea celastroides* K. (Jiménez-Estrada *et al.*, 2000 ▸) and *Hyppocratea volubilis* L. (Alvarenga *et al.*, 2000 ▸). In these publications, the crystal structure of the adduct was reported as a solvate of di­chloro­methane and toluene, respectively. The X-ray analysis showed that the Diels–Alder adduct was integrated by the triterpene ochraceolide A and a theoretical diterpene, in which the former seems to have acted as dienophile and the latter as diene in the biosynthesis. Herein the first isolation of ochraceolide A from *Elaeodendron trichotomum* (Turcz.) Lundell stem bark is reported and the crystal structure described.
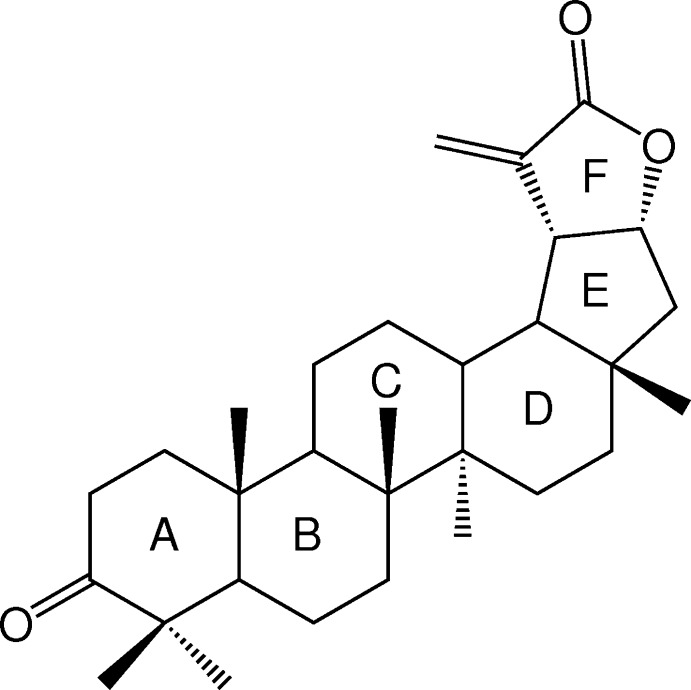


## Structural commentary   

**Figure 1:**
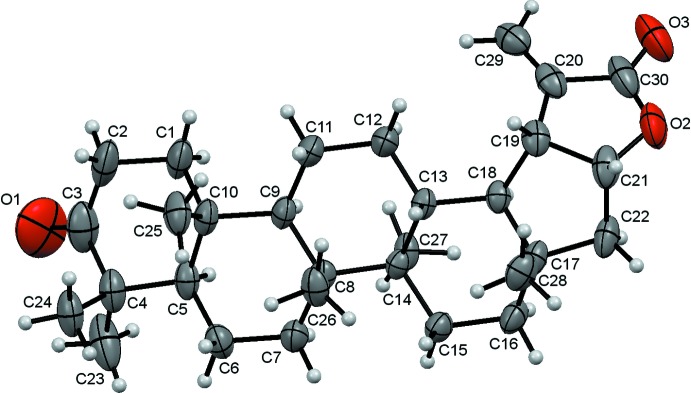
The molecular structure of the title compound with the atom labelling. Displacement ellipsoids are drawn at the 50% probability level and H atoms are shown as spheres of arbitrary radius.

The title compound has a lupane skeleton and crystallizes in the ortho­rhom­bic space group *P*2_1_2_1_2_1_ with one mol­ecule in the asymmetric unit (Fig. 1 ▸). The triterpene skeleton consists of four fused six-membered rings (*A*–*D*) and two five-membered rings (*E* and *F*). The cyclo­hexane rings are *trans*-fused and in standard chair conformations. The cyclo­pentane (C17–C19/C21/C22) ring is *trans*-fused to the triterpene *D* ring and exhibits an envelope conformation [*Q* = 0.451 (4) Å and *θ* = 356.7 (5)°] with the puckered C17 atom having the maximum deviation of 0.285 (4) Å. The α-methyl­ene γ-lactone is *cis*-fused at C19–C21 to the cyclo­pentane *E* ring and is essentially planar with a maximum deviation of 0.006 (4) Å for atom C19. The torsion angle C20—C19—C21—O2 is 0.8 (4)° and the weighted average absolute inter­nal torsion angle for the lactone ring is 0.7 (2)°

## Supra­molecular features   

**Figure 2:**
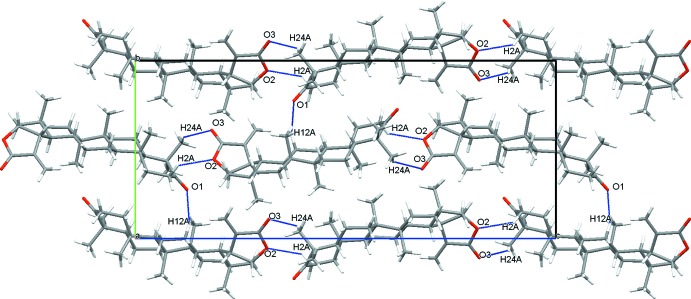
Part of the crystal structure showing hydrogen bonds as blue lines.

In the crystal, mol­ecules are linked by weak C—H⋯O hydrogen bonds (Table 1 ▸, Fig. 2 ▸). The lactone and *A* rings of adjacent mol­ecules inter­act through two hydrogen bonds (C2—H2*A*⋯O2 and C24—H24*A*⋯O3) in a head-to-tail arrangement, forming chains along [001]. These chains are further connected through a weak hydrogen bond between the oxygen of the ketone group (O1) and a methyl­ene group on the *C* ring (C12), forming an overall three-dimensional network.

## Database survey   

A search of the Cambridge Structural Database (CSD Version 5.38, update November 2016; Groom *et al.*, 2016 ▸) for α-methyl­ene γ-lactone fused to a cyclo­pentane ring gave only one entry for 6,6-dimethyl-3-methyl­ene­tetra­hydro-2*H*-cyclo­penta­[*b*]furan-2,5(3*H*)-dione (CCDC 658922; Edwards *et al.*, 2008 ▸). In both compounds, the principal supra­molecular inter­actions are C—H⋯O hydrogen bonds and the α-methyl­ene γ-lactones are *cis*-fused to the corresponding cyclo­pentane ring. However, unlike the title compound, the γ-lactone of the synthetic compound presents a twisted conformation.

## Isolation and crystallization   

*Elaeodendron trichotomum* (Turcz.) Lundell was collected from Chunchucmil, Yucatán, México (20^o^ 51.032′ N, 90^o^ 11.488′ W). A voucher specimen (JTun2328) was deposited at the Herbarium Alfredo Barrera Marín, Universidad Autónoma de Yucatán, México. Dried and milled stem bark (2100 g) was exhaustively extracted by di­chloro­methane using a Soxhlet extraction apparatus to yield 184.2 g of crude extract. A portion of the extract (100 g) was chromatographed on silica gel (40–60 µm) using a gradient elution with *n*-hexa­ne–ethyl acetate (10–100% ethyl acetate), to obtain 44 fractions. Single crystals suitable for X-ray structure analysis were obtained by slow evaporation of the mixture of solvents present in fractions 7–10 at room temperature.

## Refinement   

Crystal data, data collection and structure refinement details are summarized in Table 2 ▸. Hydrogen atoms bonded to C atoms were positioned geometrically and refined using a riding model with C—H = 0.95–1.00 Å with *U*_iso_(H) = 1.2*U*_eq_(C) or 1.5*U*_eq_(methyl C).

## Supplementary Material

Crystal structure: contains datablock(s) I. DOI: 10.1107/S2056989017012816/lh4023sup1.cif

Structure factors: contains datablock(s) I. DOI: 10.1107/S2056989017012816/lh4023Isup2.hkl

CCDC reference: 1573017

Additional supporting information:  crystallographic information; 3D view; checkCIF report

## Figures and Tables

**Table 1:** Hydrogen-bond geometry (Å, °)

*D*—H⋯*A*	*D*—H	H⋯*A*	*D*⋯*A*	*D*—H⋯*A*
C2—H2*A*⋯O2^i^	0.99	2.57	3.395 (5)	141
C12—H12*A*⋯O1^ii^	0.99	2.45	3.310 (6)	146
C24—H24*A*⋯O3^i^	0.98	2.58	3.357 (6)	137

Symmetry codes: (i) 
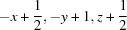
; (ii) 
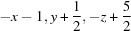
.

**Table 2:** Experimental details

Crystal data	
Chemical formula	C_30_H_44_O_3_
*M*_r_	452.65
Crystal system, space group	Orthorhombic, *P*2_1_2_1_2_1_
Temperature (K)	150
*a*, *b*, *c* (Å)	7.6131 (5), 11.7216 (7), 27.7076 (17)
*V* (Å^3^)	2472.6 (3)
*Z*	4
Radiation type	Cu *K*α
μ (mm^−1^)	0.59
Crystal size (mm)	0.36 × 0.27 × 0.25

Data collection	
Diffractometer	Bruker D8 Venture
Absorption correction	Multi-scan (*SADABS*; Krause *et al.*, 2015 ▸)
*T*_min_, *T*_max_	0.783, 0.864
No. of measured, independent and observed [*I* > 2σ(*I*)] reflections	14632, 4513, 4057
*R*_int_	0.061
(sin θ/λ)_max_ (Å^−1^)	0.603

Refinement	
*R*[*F* ^2^ > 2σ(*F* ^2^)], *wR*(*F* ^2^), *S*	0.061, 0.164, 1.09
No. of reflections	4513
No. of parameters	304
H-atom treatment	H-atom parameters constrained
Δρ_max_, Δρ_min_ (e Å^−3^)	0.28, −0.19
Absolute structure	Flack *x* determined using 1515 quotients [(*I* ^+^)−(*I* ^−^)]/[(*I* ^+^)+(*I* ^−^)] (Parsons *et al.*, 2013 ▸).
Absolute structure parameter	0.2 (3)

Computer programs: *APEX3* and *SAINT* (Bruker, 2014 ▸), *SHELXS2014* (Bruker, 2014 ▸), *SHELXL2014/7* (Sheldrick, 2015 ▸), *Mercury* (Macrae *et al.*, 2006 ▸), *PLATON* (Spek, 2009 ▸) and *publCIF* (Westrip, 2010 ▸).

## supplementary crystallographic information

### Crystal data

**Table:**

C_30_H_44_O_3_	*D*_x_ = 1.216 Mg m^−^^3^
*M**_r_* = 452.65	Cu *K*α radiation, λ = 1.54178 Å
Orthorhombic, *P*2_1_2_1_2_1_	Cell parameters from 9889 reflections
*a* = 7.6131 (5) Å	θ = 3.2–68.3°
*b* = 11.7216 (7) Å	µ = 0.59 mm^−^^1^
*c* = 27.7076 (17) Å	*T* = 150 K
*V* = 2472.6 (3) Å^3^	Prism, colourless
*Z* = 4	0.36 × 0.27 × 0.25 mm
*F*(000) = 992	

### Data collection

**Table:**

Bruker D8 Venture diffractometer	4513 independent reflections
Radiation source: micro-focus X-ray source	4057 reflections with *I* > 2σ(*I*)
Detector resolution: 52.0833 pixels mm^-1^	*R*_int_ = 0.061
ω–scans	θ_max_ = 68.3°, θ_min_ = 3.2°
Absorption correction: multi-scan (SADABS; Krause *et al.*, 2015)	*h* = −9→8
*T*_min_ = 0.783, *T*_max_ = 0.864	*k* = −13→14
14632 measured reflections	*l* = −33→33

### Refinement

**Table:**

Refinement on *F*^2^	Hydrogen site location: inferred from neighbouring sites
Least-squares matrix: full	H-atom parameters constrained
*R*[*F*^2^ > 2σ(*F*^2^)] = 0.061	*w* = 1/[σ^2^(*F*_o_^2^) + (0.0789*P*)^2^ + 0.8039*P*] where *P* = (*F*_o_^2^ + 2*F*_c_^2^)/3
*wR*(*F*^2^) = 0.164	(Δ/σ)_max_ < 0.001
*S* = 1.09	Δρ_max_ = 0.28 e Å^−^^3^
4513 reflections	Δρ_min_ = −0.19 e Å^−^^3^
304 parameters	Absolute structure: Flack *x* determined using 1515 quotients [(*I*^+^)-(*I*^-^)]/[(*I*^+^)+(*I*^-^)] (Parsons *et al.*, 2013).
0 restraints	Absolute structure parameter: 0.2 (3)

### Special details

**Table:**

Geometry. All esds (except the esd in the dihedral angle between two l.s. planes) are estimated using the full covariance matrix. The cell esds are taken into account individually in the estimation of esds in distances, angles and torsion angles; correlations between esds in cell parameters are only used when they are defined by crystal symmetry. An approximate (isotropic) treatment of cell esds is used for estimating esds involving l.s. planes.

### Fractional atomic coordinates and isotropic or equivalent isotropic displacement parameters (Å^2^)

**Table:**

	*x*	*y*	*z*	*U*_iso_*/*U*_eq_	
O1	0.7000 (9)	0.2747 (4)	1.1228 (2)	0.127 (2)	
O2	0.2109 (5)	0.5554 (3)	0.68677 (10)	0.0712 (10)	
O3	0.0663 (6)	0.3910 (4)	0.68208 (13)	0.0865 (12)	
C1	0.4287 (7)	0.3773 (4)	1.02722 (13)	0.0568 (11)	
H1A	0.3012	0.3872	1.0216	0.068*	
H1B	0.4687	0.3118	1.0075	0.068*	
C2	0.4602 (8)	0.3508 (4)	1.08081 (14)	0.0692 (14)	
H2A	0.3999	0.4094	1.1005	0.083*	
H2B	0.4059	0.2762	1.0884	0.083*	
C3	0.6490 (8)	0.3471 (4)	1.09523 (16)	0.0674 (14)	
C4	0.7745 (6)	0.4361 (3)	1.07509 (13)	0.0498 (10)	
C5	0.7231 (6)	0.4658 (3)	1.02182 (11)	0.0415 (8)	
H5	0.7540	0.3957	1.0031	0.050*	
C6	0.8379 (6)	0.5589 (3)	0.99999 (13)	0.0482 (9)	
H6A	0.9620	0.5455	1.0091	0.058*	
H6B	0.8022	0.6339	1.0131	0.058*	
C7	0.8213 (5)	0.5604 (3)	0.94485 (12)	0.0443 (8)	
H7A	0.8673	0.4876	0.9319	0.053*	
H7B	0.8953	0.6228	0.9319	0.053*	
C8	0.6322 (5)	0.5770 (3)	0.92684 (11)	0.0347 (7)	
C9	0.5071 (5)	0.4933 (3)	0.95387 (10)	0.0372 (8)	
H9	0.5416	0.4159	0.9420	0.045*	
C10	0.5260 (5)	0.4859 (3)	1.01054 (11)	0.0420 (8)	
C11	0.3177 (5)	0.5085 (4)	0.93680 (12)	0.0460 (9)	
H11A	0.2746	0.5839	0.9478	0.055*	
H11B	0.2436	0.4494	0.9522	0.055*	
C12	0.2954 (5)	0.5006 (3)	0.88168 (12)	0.0424 (8)	
H12A	0.3119	0.4204	0.8715	0.051*	
H12B	0.1743	0.5235	0.8730	0.051*	
C13	0.4255 (5)	0.5761 (3)	0.85450 (11)	0.0326 (7)	
H13	0.3992	0.6569	0.8634	0.039*	
C14	0.6178 (4)	0.5509 (3)	0.87041 (11)	0.0321 (7)	
C15	0.7510 (5)	0.6247 (3)	0.84160 (12)	0.0410 (8)	
H15A	0.7459	0.7039	0.8539	0.049*	
H15B	0.8708	0.5953	0.8478	0.049*	
C16	0.7197 (5)	0.6269 (4)	0.78673 (12)	0.0457 (9)	
H16A	0.7982	0.6842	0.7718	0.055*	
H16B	0.7496	0.5514	0.7730	0.055*	
C17	0.5299 (5)	0.6555 (3)	0.77439 (12)	0.0405 (8)	
C18	0.4109 (4)	0.5670 (3)	0.79938 (11)	0.0336 (7)	
H18	0.4567	0.4900	0.7903	0.040*	
C19	0.2307 (5)	0.5807 (3)	0.77408 (12)	0.0415 (8)	
H19	0.1522	0.6334	0.7925	0.050*	
C20	0.1359 (5)	0.4732 (3)	0.76020 (13)	0.0460 (9)	
C21	0.2797 (6)	0.6321 (4)	0.72350 (13)	0.0522 (10)	
H21	0.2275	0.7099	0.7198	0.063*	
C22	0.4785 (6)	0.6384 (4)	0.72141 (12)	0.0504 (10)	
H22A	0.5178	0.7034	0.7013	0.060*	
H22B	0.5291	0.5670	0.7083	0.060*	
C30	0.1290 (6)	0.4657 (5)	0.70646 (16)	0.0625 (13)	
C29	0.0717 (5)	0.3894 (4)	0.78608 (15)	0.0525 (10)	
H29A	0.0215	0.3248	0.7706	0.063*	
H29B	0.0754	0.3932	0.8203	0.063*	
C26	0.5821 (6)	0.7027 (3)	0.93685 (12)	0.0470 (9)	
H26A	0.6189	0.7235	0.9696	0.071*	
H26B	0.4546	0.7118	0.9339	0.071*	
H26C	0.6411	0.7523	0.9134	0.071*	
C28	0.4863 (6)	0.7796 (3)	0.78893 (14)	0.0491 (9)	
H28A	0.5315	0.7947	0.8214	0.074*	
H28B	0.3587	0.7905	0.7886	0.074*	
H28C	0.5410	0.8324	0.7660	0.074*	
C27	0.6651 (5)	0.4251 (3)	0.85928 (11)	0.0382 (8)	
H27A	0.6767	0.4150	0.8243	0.057*	
H27B	0.5721	0.3750	0.8715	0.057*	
H27C	0.7765	0.4057	0.8750	0.057*	
C25	0.4458 (7)	0.5894 (4)	1.03594 (13)	0.0546 (11)	
H25A	0.3345	0.6096	1.0203	0.082*	
H25B	0.5271	0.6540	1.0338	0.082*	
H25C	0.4243	0.5709	1.0699	0.082*	
C24	0.7708 (7)	0.5395 (4)	1.10971 (13)	0.0584 (12)	
H24A	0.6501	0.5677	1.1127	0.088*	
H24B	0.8460	0.6002	1.0969	0.088*	
H24C	0.8140	0.5161	1.1415	0.088*	
C23	0.9581 (8)	0.3866 (5)	1.07703 (17)	0.0815 (18)	
H23A	0.9834	0.3605	1.1099	0.122*	
H23B	1.0434	0.4453	1.0677	0.122*	
H23C	0.9665	0.3220	1.0547	0.122*	

### Atomic displacement parameters (Å^2^)

**Table:**

	*U*^11^	*U*^22^	*U*^33^	*U*^12^	*U*^13^	*U*^23^
O1	0.166 (5)	0.085 (3)	0.131 (4)	0.003 (3)	−0.022 (4)	0.066 (3)
O2	0.079 (2)	0.100 (3)	0.0340 (13)	0.015 (2)	−0.0172 (15)	0.0057 (15)
O3	0.090 (3)	0.108 (3)	0.062 (2)	0.011 (2)	−0.0361 (19)	−0.023 (2)
C1	0.084 (3)	0.058 (2)	0.0289 (17)	−0.010 (2)	0.0078 (19)	0.0033 (16)
C2	0.116 (4)	0.061 (3)	0.0311 (19)	−0.014 (3)	0.013 (2)	0.0049 (18)
C3	0.115 (4)	0.041 (2)	0.046 (2)	0.011 (2)	−0.007 (3)	0.0053 (18)
C4	0.076 (3)	0.0434 (19)	0.0296 (16)	0.0128 (19)	−0.0052 (17)	0.0002 (14)
C5	0.064 (2)	0.0337 (16)	0.0270 (15)	0.0099 (16)	−0.0007 (15)	−0.0032 (13)
C6	0.057 (2)	0.050 (2)	0.0371 (18)	−0.0026 (18)	−0.0093 (17)	0.0000 (15)
C7	0.050 (2)	0.049 (2)	0.0336 (16)	−0.0053 (17)	−0.0016 (16)	0.0048 (15)
C8	0.0472 (19)	0.0259 (14)	0.0312 (15)	−0.0022 (13)	0.0003 (14)	−0.0001 (12)
C9	0.050 (2)	0.0376 (17)	0.0242 (14)	−0.0016 (15)	0.0050 (14)	−0.0011 (12)
C10	0.060 (2)	0.0402 (18)	0.0253 (14)	0.0005 (17)	0.0048 (15)	−0.0029 (13)
C11	0.045 (2)	0.061 (2)	0.0320 (16)	−0.0069 (17)	0.0054 (15)	0.0050 (16)
C12	0.0439 (19)	0.050 (2)	0.0329 (16)	−0.0072 (16)	0.0017 (15)	0.0037 (15)
C13	0.0406 (17)	0.0297 (15)	0.0274 (14)	0.0047 (13)	0.0028 (13)	0.0006 (12)
C14	0.0400 (17)	0.0275 (15)	0.0288 (14)	0.0006 (13)	0.0038 (13)	0.0023 (11)
C15	0.0418 (19)	0.0447 (18)	0.0364 (17)	−0.0028 (15)	0.0029 (14)	0.0093 (14)
C16	0.047 (2)	0.054 (2)	0.0360 (18)	0.0031 (17)	0.0089 (15)	0.0156 (16)
C17	0.049 (2)	0.0415 (18)	0.0305 (16)	0.0104 (15)	0.0075 (15)	0.0103 (14)
C18	0.0394 (18)	0.0333 (16)	0.0280 (15)	0.0087 (14)	−0.0002 (13)	0.0013 (12)
C19	0.0452 (19)	0.0445 (18)	0.0348 (16)	0.0176 (15)	−0.0005 (15)	0.0002 (14)
C20	0.0397 (19)	0.055 (2)	0.0432 (19)	0.0165 (17)	−0.0098 (16)	−0.0102 (17)
C21	0.063 (2)	0.058 (2)	0.0356 (18)	0.024 (2)	−0.0016 (17)	0.0059 (16)
C22	0.062 (2)	0.061 (2)	0.0288 (17)	0.0212 (19)	0.0064 (16)	0.0122 (16)
C30	0.057 (3)	0.087 (3)	0.044 (2)	0.022 (3)	−0.021 (2)	−0.014 (2)
C29	0.043 (2)	0.060 (2)	0.055 (2)	0.0029 (18)	−0.0046 (18)	−0.0162 (19)
C26	0.075 (3)	0.0307 (17)	0.0350 (17)	−0.0006 (17)	−0.0062 (18)	−0.0047 (13)
C28	0.063 (3)	0.0358 (18)	0.049 (2)	0.0035 (17)	0.0063 (19)	0.0128 (16)
C27	0.052 (2)	0.0354 (17)	0.0271 (14)	0.0116 (15)	−0.0014 (14)	0.0004 (13)
C25	0.070 (3)	0.062 (2)	0.0316 (17)	0.020 (2)	0.0030 (18)	−0.0088 (17)
C24	0.093 (3)	0.052 (2)	0.0298 (16)	0.011 (2)	−0.0087 (19)	−0.0049 (16)
C23	0.106 (4)	0.095 (4)	0.044 (2)	0.049 (4)	−0.019 (3)	−0.010 (2)

### Geometric parameters (Å, º)

**Table:**

O1—C3	1.206 (6)	C14—C15	1.553 (5)
O2—C30	1.338 (7)	C15—C16	1.539 (5)
O2—C21	1.455 (6)	C15—H15A	0.9900
O3—C30	1.205 (6)	C15—H15B	0.9900
C1—C2	1.536 (5)	C16—C17	1.522 (5)
C1—C10	1.544 (6)	C16—H16A	0.9900
C1—H1A	0.9900	C16—H16B	0.9900
C1—H1B	0.9900	C17—C22	1.532 (5)
C2—C3	1.492 (8)	C17—C18	1.542 (5)
C2—H2A	0.9900	C17—C28	1.545 (5)
C2—H2B	0.9900	C18—C19	1.549 (5)
C3—C4	1.521 (7)	C18—H18	1.0000
C4—C23	1.515 (7)	C19—C20	1.502 (6)
C4—C24	1.546 (5)	C19—C21	1.570 (5)
C4—C5	1.566 (4)	C19—H19	1.0000
C5—C6	1.523 (6)	C20—C29	1.311 (6)
C5—C10	1.550 (6)	C20—C30	1.492 (5)
C5—H5	1.0000	C21—C22	1.517 (6)
C6—C7	1.533 (5)	C21—H21	1.0000
C6—H6A	0.9900	C22—H22A	0.9900
C6—H6B	0.9900	C22—H22B	0.9900
C7—C8	1.536 (5)	C29—H29A	0.9500
C7—H7A	0.9900	C29—H29B	0.9500
C7—H7B	0.9900	C26—H26A	0.9800
C8—C26	1.546 (5)	C26—H26B	0.9800
C8—C9	1.559 (5)	C26—H26C	0.9800
C8—C14	1.597 (4)	C28—H28A	0.9800
C9—C11	1.528 (5)	C28—H28B	0.9800
C9—C10	1.579 (4)	C28—H28C	0.9800
C9—H9	1.0000	C27—H27A	0.9800
C10—C25	1.530 (5)	C27—H27B	0.9800
C11—C12	1.539 (4)	C27—H27C	0.9800
C11—H11A	0.9900	C25—H25A	0.9800
C11—H11B	0.9900	C25—H25B	0.9800
C12—C13	1.527 (5)	C25—H25C	0.9800
C12—H12A	0.9900	C24—H24A	0.9800
C12—H12B	0.9900	C24—H24B	0.9800
C13—C18	1.535 (4)	C24—H24C	0.9800
C13—C14	1.557 (5)	C23—H23A	0.9800
C13—H13	1.0000	C23—H23B	0.9800
C14—C27	1.549 (4)	C23—H23C	0.9800
			
C30—O2—C21	111.6 (3)	C16—C15—H15B	108.6
C2—C1—C10	112.4 (4)	C14—C15—H15B	108.6
C2—C1—H1A	109.1	H15A—C15—H15B	107.6
C10—C1—H1A	109.1	C17—C16—C15	111.9 (3)
C2—C1—H1B	109.1	C17—C16—H16A	109.2
C10—C1—H1B	109.1	C15—C16—H16A	109.2
H1A—C1—H1B	107.8	C17—C16—H16B	109.2
C3—C2—C1	114.5 (4)	C15—C16—H16B	109.2
C3—C2—H2A	108.6	H16A—C16—H16B	107.9
C1—C2—H2A	108.6	C16—C17—C22	115.4 (3)
C3—C2—H2B	108.6	C16—C17—C18	108.0 (3)
C1—C2—H2B	108.6	C22—C17—C18	101.1 (3)
H2A—C2—H2B	107.6	C16—C17—C28	110.7 (4)
O1—C3—C2	120.0 (6)	C22—C17—C28	108.5 (3)
O1—C3—C4	120.9 (6)	C18—C17—C28	112.9 (3)
C2—C3—C4	119.1 (4)	C13—C18—C17	111.0 (3)
C23—C4—C3	107.7 (4)	C13—C18—C19	120.5 (3)
C23—C4—C24	107.2 (4)	C17—C18—C19	104.3 (3)
C3—C4—C24	107.4 (4)	C13—C18—H18	106.8
C23—C4—C5	110.4 (3)	C17—C18—H18	106.8
C3—C4—C5	110.0 (4)	C19—C18—H18	106.8
C24—C4—C5	113.9 (3)	C20—C19—C18	117.0 (3)
C6—C5—C10	111.5 (3)	C20—C19—C21	101.9 (3)
C6—C5—C4	113.0 (3)	C18—C19—C21	103.5 (3)
C10—C5—C4	117.7 (3)	C20—C19—H19	111.2
C6—C5—H5	104.3	C18—C19—H19	111.2
C10—C5—H5	104.3	C21—C19—H19	111.2
C4—C5—H5	104.3	C29—C20—C30	119.2 (4)
C5—C6—C7	110.9 (3)	C29—C20—C19	131.9 (3)
C5—C6—H6A	109.5	C30—C20—C19	108.8 (4)
C7—C6—H6A	109.5	O2—C21—C22	111.3 (3)
C5—C6—H6B	109.5	O2—C21—C19	107.6 (4)
C7—C6—H6B	109.5	C22—C21—C19	106.9 (3)
H6A—C6—H6B	108.0	O2—C21—H21	110.3
C6—C7—C8	113.7 (3)	C22—C21—H21	110.3
C6—C7—H7A	108.8	C19—C21—H21	110.3
C8—C7—H7A	108.8	C21—C22—C17	103.0 (3)
C6—C7—H7B	108.8	C21—C22—H22A	111.2
C8—C7—H7B	108.8	C17—C22—H22A	111.2
H7A—C7—H7B	107.7	C21—C22—H22B	111.2
C7—C8—C26	107.0 (3)	C17—C22—H22B	111.2
C7—C8—C9	109.7 (3)	H22A—C22—H22B	109.1
C26—C8—C9	111.3 (3)	O3—C30—O2	121.8 (4)
C7—C8—C14	111.0 (3)	O3—C30—C20	128.1 (5)
C26—C8—C14	110.0 (3)	O2—C30—C20	110.1 (4)
C9—C8—C14	107.9 (2)	C20—C29—H29A	120.0
C11—C9—C8	110.7 (3)	C20—C29—H29B	120.0
C11—C9—C10	113.6 (3)	H29A—C29—H29B	120.0
C8—C9—C10	117.1 (3)	C8—C26—H26A	109.5
C11—C9—H9	104.6	C8—C26—H26B	109.5
C8—C9—H9	104.6	H26A—C26—H26B	109.5
C10—C9—H9	104.6	C8—C26—H26C	109.5
C25—C10—C1	108.9 (3)	H26A—C26—H26C	109.5
C25—C10—C5	114.5 (3)	H26B—C26—H26C	109.5
C1—C10—C5	106.2 (3)	C17—C28—H28A	109.5
C25—C10—C9	112.2 (3)	C17—C28—H28B	109.5
C1—C10—C9	107.4 (3)	H28A—C28—H28B	109.5
C5—C10—C9	107.3 (3)	C17—C28—H28C	109.5
C9—C11—C12	113.8 (3)	H28A—C28—H28C	109.5
C9—C11—H11A	108.8	H28B—C28—H28C	109.5
C12—C11—H11A	108.8	C14—C27—H27A	109.5
C9—C11—H11B	108.8	C14—C27—H27B	109.5
C12—C11—H11B	108.8	H27A—C27—H27B	109.5
H11A—C11—H11B	107.7	C14—C27—H27C	109.5
C13—C12—C11	112.5 (3)	H27A—C27—H27C	109.5
C13—C12—H12A	109.1	H27B—C27—H27C	109.5
C11—C12—H12A	109.1	C10—C25—H25A	109.5
C13—C12—H12B	109.1	C10—C25—H25B	109.5
C11—C12—H12B	109.1	H25A—C25—H25B	109.5
H12A—C12—H12B	107.8	C10—C25—H25C	109.5
C12—C13—C18	113.8 (3)	H25A—C25—H25C	109.5
C12—C13—C14	111.1 (3)	H25B—C25—H25C	109.5
C18—C13—C14	109.7 (3)	C4—C24—H24A	109.5
C12—C13—H13	107.3	C4—C24—H24B	109.5
C18—C13—H13	107.3	H24A—C24—H24B	109.5
C14—C13—H13	107.3	C4—C24—H24C	109.5
C27—C14—C15	106.0 (3)	H24A—C24—H24C	109.5
C27—C14—C13	110.0 (3)	H24B—C24—H24C	109.5
C15—C14—C13	111.3 (3)	C4—C23—H23A	109.5
C27—C14—C8	111.2 (2)	C4—C23—H23B	109.5
C15—C14—C8	110.6 (3)	H23A—C23—H23B	109.5
C13—C14—C8	107.8 (2)	C4—C23—H23C	109.5
C16—C15—C14	114.6 (3)	H23A—C23—H23C	109.5
C16—C15—H15A	108.6	H23B—C23—H23C	109.5
C14—C15—H15A	108.6		
			
C10—C1—C2—C3	−52.1 (6)	C18—C13—C14—C8	172.8 (2)
C1—C2—C3—O1	−139.3 (5)	C7—C8—C14—C27	62.4 (4)
C1—C2—C3—C4	41.2 (6)	C26—C8—C14—C27	−179.3 (3)
O1—C3—C4—C23	24.3 (6)	C9—C8—C14—C27	−57.8 (4)
C2—C3—C4—C23	−156.2 (4)	C7—C8—C14—C15	−55.1 (3)
O1—C3—C4—C24	−90.8 (6)	C26—C8—C14—C15	63.2 (4)
C2—C3—C4—C24	88.7 (5)	C9—C8—C14—C15	−175.3 (3)
O1—C3—C4—C5	144.7 (5)	C7—C8—C14—C13	−176.9 (3)
C2—C3—C4—C5	−35.8 (5)	C26—C8—C14—C13	−58.7 (4)
C23—C4—C5—C6	−64.0 (5)	C9—C8—C14—C13	62.9 (3)
C3—C4—C5—C6	177.2 (3)	C27—C14—C15—C16	72.7 (4)
C24—C4—C5—C6	56.6 (5)	C13—C14—C15—C16	−46.9 (4)
C23—C4—C5—C10	163.7 (4)	C8—C14—C15—C16	−166.6 (3)
C3—C4—C5—C10	44.9 (4)	C14—C15—C16—C17	50.6 (4)
C24—C4—C5—C10	−75.7 (5)	C15—C16—C17—C22	−169.3 (3)
C10—C5—C6—C7	−61.6 (4)	C15—C16—C17—C18	−57.2 (4)
C4—C5—C6—C7	163.1 (3)	C15—C16—C17—C28	66.9 (4)
C5—C6—C7—C8	57.3 (4)	C12—C13—C18—C17	173.2 (3)
C6—C7—C8—C26	72.4 (4)	C14—C13—C18—C17	−61.6 (3)
C6—C7—C8—C9	−48.5 (4)	C12—C13—C18—C19	51.0 (4)
C6—C7—C8—C14	−167.6 (3)	C14—C13—C18—C19	176.2 (3)
C7—C8—C9—C11	179.7 (3)	C16—C17—C18—C13	64.1 (4)
C26—C8—C9—C11	61.4 (4)	C22—C17—C18—C13	−174.3 (3)
C14—C8—C9—C11	−59.3 (3)	C28—C17—C18—C13	−58.6 (4)
C7—C8—C9—C10	47.2 (4)	C16—C17—C18—C19	−164.7 (3)
C26—C8—C9—C10	−71.1 (4)	C22—C17—C18—C19	−43.1 (3)
C14—C8—C9—C10	168.2 (3)	C28—C17—C18—C19	72.6 (4)
C2—C1—C10—C25	−66.7 (5)	C13—C18—C19—C20	−98.8 (4)
C2—C1—C10—C5	57.0 (5)	C17—C18—C19—C20	135.8 (3)
C2—C1—C10—C9	171.6 (4)	C13—C18—C19—C21	150.0 (3)
C6—C5—C10—C25	−68.7 (4)	C17—C18—C19—C21	24.7 (3)
C4—C5—C10—C25	64.3 (4)	C18—C19—C20—C29	63.3 (5)
C6—C5—C10—C1	171.1 (3)	C21—C19—C20—C29	175.3 (4)
C4—C5—C10—C1	−56.0 (4)	C18—C19—C20—C30	−113.1 (3)
C6—C5—C10—C9	56.5 (4)	C21—C19—C20—C30	−1.0 (4)
C4—C5—C10—C9	−170.6 (3)	C30—O2—C21—C22	116.4 (4)
C11—C9—C10—C25	−55.7 (4)	C30—O2—C21—C19	−0.3 (4)
C8—C9—C10—C25	75.5 (4)	C20—C19—C21—O2	0.8 (4)
C11—C9—C10—C1	64.0 (4)	C18—C19—C21—O2	122.7 (3)
C8—C9—C10—C1	−164.8 (3)	C20—C19—C21—C22	−118.8 (4)
C11—C9—C10—C5	177.8 (3)	C18—C19—C21—C22	3.1 (4)
C8—C9—C10—C5	−51.0 (4)	O2—C21—C22—C17	−146.9 (3)
C8—C9—C11—C12	53.1 (4)	C19—C21—C22—C17	−29.8 (4)
C10—C9—C11—C12	−172.7 (3)	C16—C17—C22—C21	160.8 (3)
C9—C11—C12—C13	−49.7 (4)	C18—C17—C22—C21	44.7 (4)
C11—C12—C13—C18	178.0 (3)	C28—C17—C22—C21	−74.3 (4)
C11—C12—C13—C14	53.6 (4)	C21—O2—C30—O3	−178.3 (4)
C12—C13—C14—C27	60.9 (3)	C21—O2—C30—C20	−0.4 (5)
C18—C13—C14—C27	−65.8 (3)	C29—C20—C30—O3	1.8 (7)
C12—C13—C14—C15	178.1 (3)	C19—C20—C30—O3	178.7 (4)
C18—C13—C14—C15	51.4 (3)	C29—C20—C30—O2	−176.0 (4)
C12—C13—C14—C8	−60.5 (3)	C19—C20—C30—O2	0.9 (5)

### Hydrogen-bond geometry (Å, º)

**Table:**

*D*—H···*A*	*D*—H	H···*A*	*D*···*A*	*D*—H···*A*
C2—H2*A*···O2^i^	0.99	2.57	3.395 (5)	141
C12—H12*A*···O1^ii^	0.99	2.45	3.310 (6)	146
C24—H24*A*···O3^i^	0.98	2.58	3.357 (6)	137

Symmetry codes: (i) −*x*+1/2, −*y*+1, *z*+1/2; (ii) −*x*−1, *y*+1/2, −*z*+5/2.

## References

[bb1] Alvarenga, N. L., Ferro, E. A., Ravelo, A. G., Kennedy, M. L., Maestro, M. A. & González, A. G. (2000). *Tetrahedron*, **56**, 3771–3774.

[bb2] Bruker (2014). *APEX2*, *SAINT* and *SADABS*. Bruker AXS Inc., Madison, Wisconsin, USA.

[bb3] Callies, O., Bedoya, L. M., Beltrán, M., Muñoz, A., Calderón, P. O., Osorio, A. A., Jiménez, I. A., Alcamí, J. & Bazzocchi, I. L. (2015). *J. Nat. Prod.* **78**, 1045–1055. 10.1021/np501025r 25927586

[bb4] Edwards, M. G., Kenworthy, M. N., Kitson, R. A., Scott, M. S. & Taylor, R. K. (2008). *Angew. Chem. Int. Ed.* **47**, 1935–1937. 10.1002/anie.200705329 18228235

[bb5] Groom, C. R., Bruno, I. J., Lightfoot, M. P. & Ward, S. C. (2016). *Acta Cryst.* B**72**, 171–179. 10.1107/S2052520616003954 PMC4822653 27048719

[bb6] Jiménez-Estrada, M., Reyes-Chilpa, R., Hernández-Ortega, S., Cristobal-Telésforo, E., Torres-Colín, L., Jankowski, C. K., Aumelas, A. & Van Calsteren, M. R. (2000). *Can. J. Chem.* **78**, 248–254.

[bb7] Krause, L., Herbst-Irmer, R., Sheldrick, G. M. & Stalke, D. (2015). *J. Appl. Cryst.* **48**, 3–10. 10.1107/S1600576714022985 PMC4453166 26089746

[bb8] Macrae, C. F., Edgington, P. R., McCabe, P., Pidcock, E., Shields, G. P., Taylor, R., Towler, M. & van de Streek, J. (2006). *J. Appl. Cryst.* **39**, 453–457.

[bb9] Ngassapa, O. D., Soejarto, D. D., Che, C., Pezzuto, J. M. & Farnsworth, N. R. (1991). *J. Nat. Prod.* **54**, 1353–1359. 10.1021/np50077a019 1800637

[bb10] Parsons, S., Flack, H. D. & Wagner, T. (2013). *Acta Cryst.* B**69**, 249–259. 10.1107/S2052519213010014 PMC3661305 23719469

[bb11] Sheldrick, G. M. (2015). *Acta Cryst.* A**71**, 3–8.

[bb12] Spek, A. L. (2009). *Acta Cryst.* D**65**, 148–155. 10.1107/S090744490804362X PMC2631630 19171970

[bb13] Sturm, S., Gil, R. R., Chai, H., Ngassapa, O. D., Santisuk, T., Reutrakul, V., Howe, A., Moss, M., Besterman, J. M., Yang, S., Farthing, J. E., Tait, R. M., Lewis, J. A., O’Neill, M. J., Farnsworth, N. R., Cordell, G. A., Pezzuto, J. M. & Kinghorn, A. D. (1996). *J. Nat. Prod.* **59**, 658–663. 10.1021/np960370u 8759161

[bb14] Westrip, S. P. (2010). *J. Appl. Cryst.* **43**, 920–925.

